# Changes in obsessive–compulsive symptoms during inpatient treatment of anorexia nervosa

**DOI:** 10.1186/s40337-022-00629-3

**Published:** 2022-07-18

**Authors:** Adrian Meule, Ulrich Voderholzer

**Affiliations:** 1 Department of Psychiatry and Psychotherapy, University Hospital, LMU Munich, Munich, Germany; 2 Schoen Clinic Roseneck, Am Roseneck 6, 83209 Prien am Chiemsee, Germany; 3 Department of Psychiatry and Psychotherapy, Faculty of Medicine, Medical Center, University of Freiburg, Freiburg, Germany

**Keywords:** Anorexia nervosa, Obsessive–compulsive disorder, Comorbidity, Body mass index, Inpatient treatment

## Abstract

**Background:**

Obsessive–compulsive disorder (OCD) is one of the most prevalent comorbidities in anorexia nervosa (AN). As AN is a severe, life-threatening condition, reducing obsessive–compulsive symptomatology is not the primary objective during treatment of AN and, thus, these symptoms may remain unchanged or may even increase in terms of a “symptom shift”.

**Methods:**

In this retrospective analysis, we examined clinical records of 149 adolescents (*n* = 96, 64%) and adults (*n* = 53, 36%) with AN (6 males, 4%) who received inpatient treatment and completed the Obsessive–Compulsive Inventory–Revised at admission and discharge.

**Results:**

Obsessive–compulsive symptoms decreased from admission to discharge, irrespective of whether patients had comorbid OCD or not. Within-person decreases in obsessive–compulsive symptoms weakly correlated with increases in body weight.

**Conclusions:**

These results indicate that obsessive–compulsive symptoms decrease during inpatient treatment of AN although they are not primarily targeted during treatment. Furthermore, these improvements seem to be associated with general improvements in AN symptomatology, suggesting the absence of a “symptom shift”. Yet, effect sizes were small and obsessive–compulsive symptoms were still clinically elevated in patients with comorbid OCD at discharge, suggesting that these patients need OCD-specific, psychotherapeutic aftercare.

## Plain English summary

This study shows that obsessive–compulsive symptoms decrease during inpatient treatment of anorexia nervosa. That is, there is no “symptom shift”, which refers to the idea that a reduction of anorexia nervosa symptoms may result in an increase in other symptoms. However, obsessive–compulsive symptoms should still be targeted during psychotherapeutic aftercare in patients who show obsessive–compulsive tendencies.

## Introduction

With a prevalence of about 10–20%, obsessive–compulsive disorder (OCD) is one of the most prevalent comorbidities in anorexia nervosa (AN; [1]). Besides phenomenological overlaps between the two conditions (e.g., ritualized behavior, cognitive rigidity), there is also a substantial shared genetic basis [2, 3]. Yet, as AN is a severe, life-threatening condition, reducing obsessive–compulsive symptomatology is not the primary objective during treatment of AN. Specifically, treatment of AN focuses on weight restoration along with addressing AN symptoms such as restrictive eating, weight and shape concerns, or compulsive exercise [4]. Although less severe cases of AN are treated in out patient or daypatient settings, more severe cases or those who do not respond to these treatments require inpatient treatment [5, 6]. There is a plethora of studies showing that inpatient treatment leads to a substantial gain in body weight and reductions in other AN symptoms in both adolescents and adults (e.g., [7–11]). However, few studies have examined if inpatient treatment also leads to decreases in obsessive–compulsive symptoms, if these symptoms remain unchanged or if they even increase in terms of a “symptom shift” [12].

In a sample with mixed eating disorder diagnoses (that only included 10 AN patients with and 17 AN patients without comorbid OCD), Thiel and colleagues [13] reported that obsessive–compulsive symptoms decreased from psychodynamic inpatient treatment to 30-months follow up only in those with comorbid OCD but not in those without comorbid OCD. The absence of changes in obsessive–compulsive symptoms in the latter group may be explained by the fact that they already had relatively low scores at admission. Furthermore, larger improvements in eating disorder symptoms related to larger improvements in obsessive–compulsive symptoms in this study. In contrast, Mattar and colleagues [14] reported no significant changes in obsessive–compulsive symptoms from admission to discharge in a sample of 24 inpatients with AN. Yet, Lee and colleagues [15] reported significant decreases in obsessive–compulsive symptoms from pre- to post-treatment in a sample with mixed eating disorder diagnoses at a residential treatment facility. Most recently, Pleplé and colleagues [16] reported significant decreases in obsessive–compulsive symptoms from admission to discharge in a large sample (*n* = 167) of inpatients with AN.

To summarize these four studies, two found that obsessive–compulsive symptoms decreased during treatment, one did not find that obsessive–compulsive symptoms decreased during treatment, and one found that obsessive–compulsive symptoms only decreased in those with comorbid OCD. Thus, although there is some evidence suggesting that obsessive–compulsive symptoms decrease during eating disorder treatment, findings are rather inconsistent. In addition, these findings were derived from different samples (two with AN patients and two with mixed eating disorder diagnoses) and involved different treatments. Furthermore, only one study in a sample with mixed eating diagnoses examined whether changes in obsessive–compulsive symptoms related to changes in eating disorder symptoms. Thus, it is currently unclear if obsessive–compulsive symptoms decrease during inpatient AN treatment, whether they only (or more strongly) change in those with comorbid OCD, and whether changes in obsessive–compulsive symptoms relate to changes in AN symptomatology.

In this retrospective analysis, we analyzed clinical records from 149 inpatients with AN and examined three research questions. First, based on the findings by Pleplé and colleagues [16], we expected that obsessive–compulsive symptoms would decrease from admission to discharge. Second, based on the findings by Thiel and colleagues [13], we hypothesized that decreases in obsessive–compulsive symptoms would be larger in patients with comorbid OCD than in patients without comorbid OCD. Third, based on the findings by Thiel and colleagues [13] who found that larger reductions in obsessive–compulsive symptoms related to related to larger decreases in eating disorder symptoms, we hypothesized that larger reductions in obsessive–compulsive symptoms would also relate to larger weight gain.

## Methods

### General study description

In this retrospective study, data from patients with AN who received inpatient treatment at the Schoen Clinic Roseneck (Prien am Chiemsee, Germany) between January 2015 and September 2021 and who completed the German version [17] of the Obsessive–Compulsive Inventory–Revised (OCI–R; [18]) at admission and discharge were analyzed. The OCI–R is not part of the routine diagnostic assessment for AN patients but is completed by patients upon request by their therapists if exploratory questions suggest that there might be an obsessive–compulsive symptomatology and, therefore, further evaluation of obsessive–compulsive symptoms is deemed necessary. At the Schoen Clinic Roseneck, data from the diagnostic assessments (e.g., age, sex, body weight and height, length of stay, diagnoses, questionnaire scores) are automatically transferred to a database from which they can be exported without any identifying information (e.g., name, date of birth, place of residence) by authorized employees. Thus, accessing individual patient charts is not necessary. According to the guidelines by the institutional review board of the LMU Munich, retrospective studies conducted on already available, anonymized data are exempt from requiring ethics approval. The data of this study are available at https://osf.io/k2g95.

### Treatment description

The inpatient treatment offered at the hospital adheres to the German S3-guidelines for the treatment of AN [5, 19] in terms of admission criteria, treatment elements, and therapy goals. Thus, patients received a cognitive-behavioral therapy-oriented, multimodal AN treatment that included several treatment elements such as individual psychotherapy sessions, group therapy sessions, exercise therapy, meal preparation classes, body image exposure, nutrition counseling, and food intake protocols as well as clinical management of medical complications. The treatment includes a high-calorie refeeding schedule (starting on the first day of treatment) that aims at a weight gain of 0.7–1.0 kg per week for all underweight AN patients. This schedule includes three meals per day, each having approximately 700 kcal and, thus, totaling to a daily caloric intake of approximately 2100 kcal. Meals are supervised by a nurse or therapist in earlier treatment stages. The schedule is individually tailored if patients do not finish their meals or do not show the expected weight gain by increasing portion size, adding snacks between meals, or offering sip feeds. As normalization of eating behavior is one of the therapeutic goals, patients do not receive nasogastric feeding. Patients can choose between vegetarian and non-vegetarian menus; vegan menus are not offered.

### Sample description

Between January 2015 and September 2021, 4350 cases with a primary diagnosis of full syndrome AN (ICD–10 code F50.0; *n* = 3808, 87.5%) or atypical AN (ICD–10 code F50.1; *n* = 542, 12.5%) were treated at the hospital. Of these, 486 patients (11.2%) were diagnosed with comorbid OCD (ICD–10 code F42) and 3864 patients (88.8%) had no comorbid OCD. For a subset of 149 patients, OCI–R scores were available both at admission and discharge. Of these, 132 patients (88.6%) were diagnosed with full syndrome AN and 17 had atypical AN (11.4%). One-hundred and one patients (67.8%) were diagnosed with comorbid OCD and 48 patients (32.2%) had no comorbid OCD.^1^ Six patients (4.0%) were male. Mean age was 18.6 years (*SD* = 5.88; 96 adolescents, 64.4%; 53 adults, 35.6%).^2^ Mean body mass index (BMI) was 15.2 kg/m^2^ (*SD* = 2.05) at admission and 18.0 kg/m^2^ (*SD* = 1.68) at discharge. Mean length of stay was 124 days (*SD* = 48.5).

### OCI–R

The OCI–R assesses obsessive–compulsive symptoms with 18 items. Responses are recorded on a five-point scale from 0 = *not at all* to 4 = *extremely*. Higher sum scores indicate higher obsessive–compulsive symptomatology. A score of 21 has been found to optimally discriminate between patients with OCD and persons without OCD [18]. Internal reliability (McDonald’s omega; cf. [20]) was ω = 0.864 at admission and ω = 0.891 at discharge in the current study.

### Data analyses

Changes in OCI–R scores from admission to discharge were tested with a paired samples *t*-test. Changes in OCI–R scores from admission to discharge as a function of comorbid OCD were tested with analysis of variance for repeated measures with the factors *time* (admission vs. discharge) and *group* (comorbid vs. no comorbid OCD). The within-person association between changes in OCI–R scores from admission to discharge and changes in BMI from admission to discharge was tested by computing a repeated measures correlation coefficient with the R-package *rmcorr* [21].

## Results

### Changes in obsessive−compulsive symptoms from admission to discharge

**Fig. 1:**
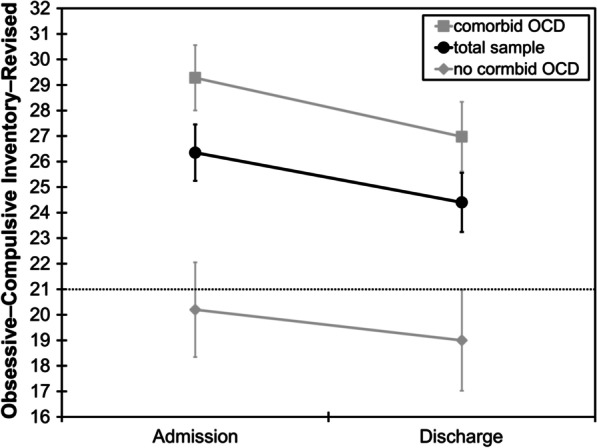
Mean sum scores of the Obsessive–Compulsive Inventory–Revised at admission and discharge in the total sample and as a function of comorbid and no comorbid obsessive–compulsive disorder (OCD). Error bars indicate standard errors of the mean. The black dotted line indicates the cut-off score of 21 that has been found to optimally discriminate between persons with and without obsessive–compulsive disorder in the study by Foa and colleagues [18]

OCI−R scores decreased from admission (*M* = 26.4, *SD* = 13.5) to discharge (*M* = 24.4, *SD* = 14.1; *t*_(148)_ = 3.20, *p* = 0.002, *d* = 0.26; Fig. 1).

### Changes in obsessive−compulsive symptoms from admission to discharge as a function of comorbid OCD

The interaction group × time was not significant (*F*_(1,147)_ = 0.72, *p* = 0.397, η_p_^2^ = 0.005), indicating that the size of changes in OCI−R scores from admission to discharge did not differ between groups (Fig. 1). Of note, although scores decreased in both groups, they were still above the cut-off score of 21 in those with comorbid OCD at discharge (Fig. 1).

### Within-person association between body weight and obsessive−compulsive symptoms across admission and discharge

**Fig. 2:**
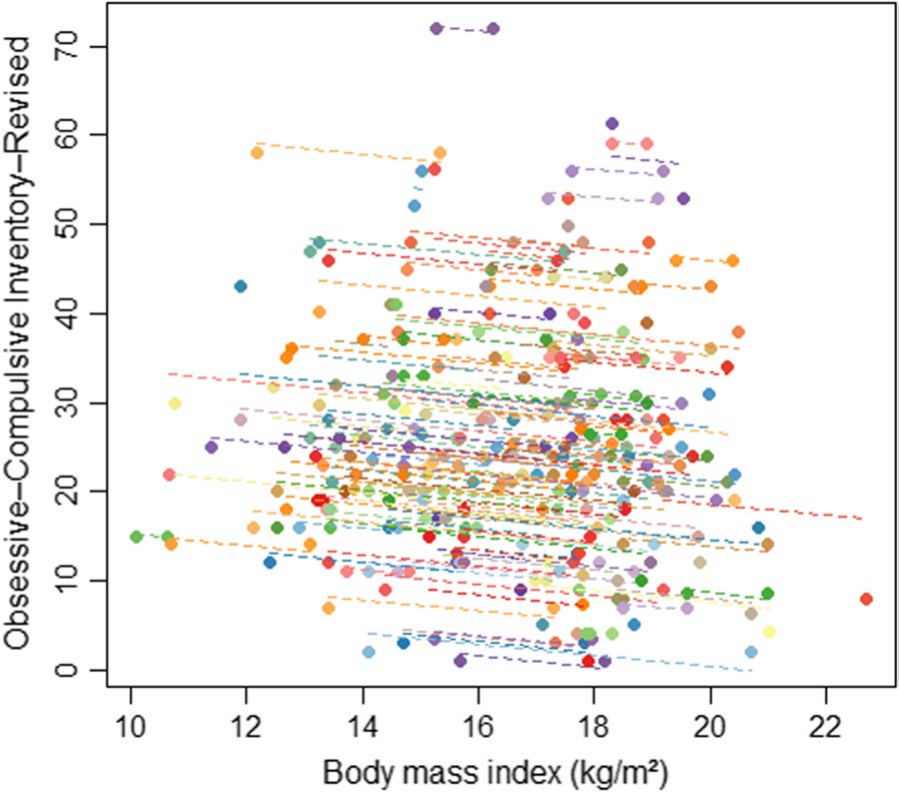
*Rmcorr* plot depicting within-person associations across admission and discharge between body mass index and scores of the Obsessive–Compulsive Inventory–Revised. Note that, in *rmcorr*, separate parallel lines are fit to the data from each person and the sign of the *rmcorr* coefficient is indicated by the direction of the common regression slope. In the rmcorr plot, each patient’s data and corresponding line are shown in different color. This is done because *rmcorr* can capture strong intra-individual relationships (here: between body weight and obsessive–compulsive symptoms across the two measurement time points) that are missed by using averaged data. As can be seen by the negative *rmcorr* coefficient (*r*_*rm*_ =  − .27) and the negative slopes of the individual fit lines, larger weight gain was associated with decreases in obsessive–compulsive symptoms

The repeated measures correlation between BMI and OCI–R scores was *r*_*rm*_ =  − 0.266 (*p* = 0.001, 95%CI[− 0.109; − 0.410]; Fig. 2), indicating that within-person weight gain was associated with decreases in obsessive–compulsive symptoms.

## Discussion

### Summary of findings

In this retrospective analysis, obsessive–compulsive symptoms significantly decreased during inpatient treatment of AN. The magnitude of this decrease was small and was similar in patients with and without comorbid OCD. Although obsessive–compulsive symptoms decreased, average scores were still clinically elevated in those with comorbid OCD at discharge. Larger decreases in obsessive–compulsive symptoms related to larger weight gain with a small effect size, suggesting that decreases in AN symptomatology do not result in a “symptom shift”, that is, do not facilitate obsessive–compulsive symptomatology.

### Clinical implications

The finding that obsessive–compulsive symptoms decrease during AN treatment and relate to general changes in AN symptoms may be partially explained by overlapping features of both conditions. For example, both AN and OCD share several phenotypic, epidemiological, and neuropsychological characteristics such as excessive habit formation, cognitive rigidity, and repetitive and ritualistic behaviors [22, 23]. Thus, treating AN symptoms may also generalize to alter obsessive–compulsive symptoms that are not related to food, eating, and body weight because of their close phenomenological—but maybe also etiological—connection [2, 3].

Intervention techniques that target these central, overlapping features of both conditions may be particularly effective in the treatment of AN. Indeed, cognitive remediation therapy—originally developed as a treatment for schizophrenia—has been adapted for eating disorders as an add-on treatment element [24]. Amongst others, it addresses cognitive rigidity by using cognitive exercises to increase set-shifting abilities (i.e., increase cognitive flexibility) and to promote a more global information processing (i.e., decrease extreme attention to details). While some pilot studies found beneficial effects of cognitive remediation therapy in patients with AN, however, overall findings have been mixed [25]. In fact, two recent randomized controlled trials did not find that cognitive remediation therapy as an add-on to treatment as usual improved clinical and cognitive outcomes when compared to an active control condition [22, 26]. Thus, further research is necessary that examines if targeting obsessive–compulsive features of AN with cognitive remediation therapy has actual benefits over and above traditional psychotherapeutic approaches such as cognitive-behavioral therapy.

### Limitations

Several factors limit interpretation of the current findings. As this was a retrospective analysis of clinical records, diagnoses were not confirmed by a structured clinical interview, which may be more precise than clinical diagnoses. However, the prevalence of comorbid OCD diagnoses (11%) matches well with prevalence rates that have been recently reported in a recent meta-analytic investigation [1], suggesting that OCD diagnoses were not under- or overestimated in the current study. Further, OCI–R scores were primarily available for those with comorbid OCD, which may have introduced a bias as the current sample was not representative of all treated cases with AN (i.e., although only 11% of all AN cases had comorbid OCD, 68% of all analyzed cases with available OCI–R scores had comorbid OCD). Finally, the current sample included both full syndrome and atypical AN patients as well as both males and females but the subgroups of atypical AN patients and males were too small to allow for testing whether changes in obsessive–compulsive symptoms differed between these groups. Yet, at least for the differentiation between full syndrome and atypical AN, it has been previously been reported that these groups do not show substantial differences in obsessive–compulsive symptoms [27].

## Conclusion

The current study shows that obsessive–compulsive symptoms decrease during inpatient treatment of AN (although these are not a primary treatment target) and these decreases are associated with increases in body weight. Of note, effect sizes were small and patients with comorbid OCD still had clinically elevated obsessive–compulsive symptomatology at discharge, suggesting that obsessive–compulsive symptoms should be targeted with OCD-specific treatment elements in psychotherapeutic aftercare.

## Data Availability

The data of this study are available at https://osf.io/k2g95.

## Footnotes

1 Note that patients with and without comorbid OCD did not differ in age (*t*_(147)_ = 1.16, *p* = .246, *d* = 0.20) and length of stay (*t*_(147)_ = 0.55, *p* = .585, *d* = 0.10). Groups also did not differ in BMI (main effect of group: *F*_(1,147)_ = 0.03, *p* = .866, η_p_^2^ < .001) and changes in BMI from admission to discharge (group × time interaction: *F*_(1,147)_ = 0.04, *p* = .835, η_p_^2^ < .001). Similarly, BMI was uncorrelated with OCI–R scores at admission (*r* = .076, *p* = .357).

2 Note that adolescents and adults did not differ in OCI–R scores (main effect of group: *F*_(1,147)_ = 0.72, *p* = .396, η_p_^2^ = .005) and changes of OCI–R scores from admission to discharge (group × time interaction: *F*_(1,147)_ < 0.01, *p* = .963, η_p_^2^ < .001). Similarly, age was uncorrelated with OCI–R scores at admission (*r* = .055, *p* = .504).
